# Odontogenesis-associated phosphoprotein (ODAPH) Promotes Ameloblast adhesion and alkaline phosphatase (ALP) expression via LAMC2/ ITGB6/TGF-β1 signaling pathway

**DOI:** 10.1371/journal.pone.0328263

**Published:** 2025-07-18

**Authors:** Mingyue Li, Jie Zhang, Shuang Xiao, Xinyang Liu, Shuai Song, Xiaoyuan Ye, Ruonan Bi, Yuguang Gao, Li Zhang

**Affiliations:** 1 School of Medicine, Qingdao Huanghai University, Qingdao, Shandong, China; 2 School of Stomatology, Binzhou Medical University, Yantai, Shandong, China; Università degli Studi della Campania, ITALY

## Abstract

Recessive hypomineralized amelogenesis imperfecta has been linked to mutations in Odontogenesis-Associated Phosphoprotein (ODAPH). Consistent with human phenotypes, *Odaph*-null mice exhibit defective enamel mineralization with ameloblast detachment from the enamel surface. To elucidate the mechanistic basis, we investigated ODAPH’s role in ameloblast adhesion and mineralization using ameloblast-lineage cells (ALCs). Key findings demonstrate that *Odaph* overexpression enhanced Lamininγ2 (LAMC2)/Integrinβ6(ITGB6)/TGF-β1/Alkaline Phosphatase(ALP) pathway activity. Notably, co-immunoprecipitation confirmed interactions between ODAPH and LAMC2. Functional analyses revealed that ITGB6 activates the TGF-β1/ALP signaling cascade. Inhibition of integrin (CWHM-12) abrogates ODAPH-mediated TGF-β1/ALP induction. TGF-β1 positively regulates both LAMC2/ITGB6 expression and ALP activity. These results establish that ODAPH orchestrates ameloblast adhesion and mineralization via the LAMC2/ITGB6/TGF-β1/ALP signaling axis.

## Introduction

Enamel represents the most highly mineralized tissue in vertebrates, with its development progressing through two distinct phases: the secretory stage and maturation stage. During the secretory stage, ameloblasts synthesize and secrete enamel matrix proteins (EMPs) into the developing enamel matrix. Subsequently, these EMPs undergo proteolytic degradation through the action of matrix metalloproteinase 20 (MMP20), facilitating the longitudinal growth of enamel crystallites [1]. During the maturation stage, a specialized basal lamina forms at the ameloblast-enamel interface, mediating cellular adhesion to the mineralizing surface [2]. Kallikrein-related peptidase-4 (KLK4) maintains high expression levels to proteolytically degrade EMPs [3]. Concurrently, ameloblasts mediate mineral ion transport to regulate enamel crystal growth in both width and thickness, ultimately achieving the requisite hardness [1,4]. Disruption of these maturation processes results in defective enamel mineralization, manifesting clinically as hypomaturation amelogenesis imperfecta characterized by soft and friable enamel [5,6].

Pathogenic mutations in Odontogenesis-associated phosphoprotein (ODAPH) are genetically linked to hypomaturation amelogenesis imperfecta [5,7]. In vitro evidence demonstrates that the phosphorylated C-terminal domain of ODAPH facilitates hydroxyapatite nucleation and modulates crystal growth, suggesting its critical role in enamel biomineralization [7,8]. Genetic ablation of *Odaph* in mice results in severe dental attrition, hypomineralized enamel, and ameloblast detachment from the enamel surface, indicating ODAPH’s essential role in maintaining basal lamina integrity during enamel maturation [9,10]. Conversely, *Odaph* overexpression leads to aberrant enamel prism organization and hypermineralization [11]. These contrasting phenotypes demonstrate ODAPH’s critical involvement in regulating ameloblast adhesion and enamel mineralization. Nevertheless, the precise molecular mechanisms underlying these processes remain to be elucidated.

The basement membrane, a specialized extracellular matrix interface between epithelial cells and underlying connective tissue, mediates critical biological processes including growth, development and cellular differentiation [12]. In odontogenesis, a unique atypical basal lamina forms between ameloblasts and the mineralizing enamel surface, representing a structural and functional specialization distinct from conventional basement membranes. The atypical basal lamina is characteristically enriched with Laminin-332, a trimeric extracellular matrix protein (α3β3γ2 chains) that mediates epithelial cell adhesion and migration [12,13]. Notably, the γ2 chain (LAMC2) serves as a defining component of this structure [14]. Our prior immunohistochemical analyses demonstrated co-localization of ODAPH with LAMC2 at the basal lamina interface, establishing ODAPH as a novel constituent of this specialized matrix [9,15]. Nevertheless, the molecular interaction between ODAPH and LAMC2 requires further elucidation.

Integrins represent a class of heterodimeric transmembrane receptors composed of α and β subunits. Among the eight mammalian Integrinβ subtypes, Integrinβ6 (ITGB6) exclusively pairs with αv subunits to form functional receptors [16,17]. This epithelial-specific integrin is prominently expressed in ameloblasts [18,19]. Biochemically, integrins interact with laminins through specific recognition of the γ chain C-terminal domains, a molecular interaction critical for cell-matrix adhesion [20]. ITGB6 serves as a critical activator of transforming growth factor-β1 (TGF-β1) through specific recognition of the RGD motif within TGF-β1 latency-associated peptides [21,22]. Genetic evidence demonstrates that RGD-to-RGE mutations phenocopy *Tgfb1*-null mice, confirming the essential role of integrin-mediated TGF-β1 activation [23,24]. As the predominant TGF-β isoform in human tissues, TGF-β1 orchestrates osteoblast differentiation and biomineralization processes through multifaceted regulatory mechanisms [25]. During odontogenesis, TGF-β1 localizes to the extracellular matrix [26,27], where it forms functional complexes with amelogenin to mediate TGF-β receptor signaling during both secretory and maturation stages of amelogenesis [28]. Importantly, physiological concentrations of TGF-β1 (0.1–1 ng/mL) significantly upregulate alkaline phosphatase (ALP) expression [29], indicating its direct role in promoting biomineralization. ALP is abundantly expressed in mineralized tissues and serves as a well-established biomarker for biomineralization processes [30,31]. This functional significance is further corroborated by the enamel hypomaturation phenotype observed in *Alpl*-deficient mice [32]. The present study systematically investigates the potential regulation of ALP expression by ODAPH through the TGF-β1 signaling pathway.

## Materials and methods

### Cell culture

The study utilized two cell lines: (1) immortalized mouse ameloblast-lineage cells (ALCs), originally isolated from neonatal C57BL/6J mouse mandibular molars by Toshihiro Sugiyama [33], cultured in DMEM/F12 medium (Meilunbio, China) supplemented with 10% fetal bovine serum (FBS; EVERY GREEN, China) and 1% penicillin-streptomycin (Meilunbio, China); and (2) HEK 293T cells maintained in DMEM (Gibco, USA) with identical supplements. All cells were incubated at 37°C in a 5% CO_2_ atmosphere.

### Lentiviral Transduction of ALC Cells

The lentiviral vector system (LV18; GenePharma, Shanghai, China) contained CMV promoter and puromycin resistance marker. We used the following constructs: (1) LV18-*Odaph*-mus (LV-*Odaph*; insert sequence: 5’-GATGTAGTCACCCCTCCTGG-3’) for *Odaph* overexpression; (2) LV18-*NC* (LV-*NC*) as negative control; (3) LV3-*Lamc2*-mus (sh*Lamc2*) for *Lamc2* knockdown; and (4) LV3-*NC* (sh*NC*) as scramble control. ALC cells were transduced with respective lentiviruses in medium containing 5 μg/mL polybrene (GenePharma, China) to enhance transduction efficiency. ODAPH and LAMC2 expression levels were quantified by Quantitative real-time PCR (qRT-PCR) and western blot at 72 hours post-transduction.

### qRT-PCR analysis

Total RNA was isolated from ALC cells using TRIzol reagent (Takara, Japan), with purity and concentration determined spectrophotometrically. Following DNase I treatment, 1 μg of total RNA was reverse-transcribed into cDNA using the PrimeScript RT Reagent Kit with gDNA Eraser (Takara, Japan) according to the manufacturer’s protocol. qRT-PCR was performed in triplicate using TB Green Premix Ex Taq (Takara, Japan) on a LightCycler 96 system (Roche, Germany). Primer sequences were as follows: *Odaph* forward 5′-GATGTAGTCACCCCTCCTGG-3′, reverse 5′-TGGGCCCTTGTTACCAGATT-3′; *Alpl* forward 5′-GCAGTATGAATTGAATCGGAACAAC-3′, reverse 5′-ATGGCCTGGTCCATCTCCAC-3′; *Lamc2* forward 5′-GCAGTGAGGCAGATAGCGTTGA-3′, reverse 5′-TGGCCACAGCGGGATTAGA-3′; *Itgb6* forward 5′-AGTGCAGAATGTGACTGCGA-3′, reverse 5′-CAGGAATCCGTGCTCACCAT-3′; *Tgfb1* forward 5′-CCATCCATGACATGAACCGG-3′, reverse 5′-ACTTCCAACCCAGGTCCTTC-3′; *Gapdh* forward 5′-TGTGTCCGTCGTGGATCTGA-3′, reverse 5′-TTGCTGTTGAAGTCGCAGGAG-3′. Gene expression levels were normalized to *Gapdh* and quantified using the 2^−ΔΔCt^ method [34].

### Western blot analysis

Protein extraction was performed using RIPA lysis buffer (Beyotime, China) supplemented with PMSF (Beyotime, China) and protease/phosphatase inhibitor cocktail (Solarbio, China). Protein concentrations were determined by BCA assay (Solarbio, China), with equal amounts (30 μg/lane) separated by 10% SDS-PAGE and electrophoretically transferred to PVDF membranes (ServiceBio, China). Following blocking with 5% skim milk, membranes were incubated with primary antibodies at 4°C overnight: anti-ODAPH (1:2000; Proteintech, #91246), anti-LAMC2 (1:2000; Invitrogen, #PA5−79578), anti-ITGB6 (1:8000; Abcam, #ab197672), anti-ALP (1:2000; Affinity, #DF6225), anti-β-Actin (1:2000; ServiceBio, #GB11001), anti-Flag (1:2000; Proteintech, #20543–1-AP), and anti-His (1:2000; Proteintech, #66005–1-Ig). After washing, membranes were probed with HRP-conjugated secondary antibody (1:5000; Beyotime, #A0208) for 1 h at room temperature. Protein bands were visualized using enhanced chemiluminescence (ECL; Beyotime) on a Tanon 4600SF imaging system.

### ALP expression and activity analysis [35]

For mineralization induction, ALC cells at 80–90% confluence were cultured in mineralization medium containing 10 mM β-glycerophosphate, 50 μg/mL ascorbic acid, and 10 nM dexamethasone (all from Meilunbio, China) for 7 or 14 days, with medium changes every 48 hours. *Alpl* mRNA levels were quantified by qRT-PCR. For ALP staining, cells were fixed with 4% paraformaldehyde (15 min), then incubated with BCIP/NBT substrate (Beyotime, China; 1 h, room temperature). After distilled water washes, images were acquired using an EVOS M5000 imaging system (Thermo Fisher Scientific).

### Cell adhesion assay

ALC cells were transduced with either LV-*Odaph* (experimental group) or LV-*NC* (control group) lentiviral vectors in the presence of 5 μg/mL polybrene to enhance transduction efficiency. Following trypsinization and reseeding, cell adhesion dynamics were assessed at 4, 8, and 12-hour intervals using phase-contrast microscopy (Olympus, Japan) on BIOFIL culture dishes (#TCP011006).

### Immunocytochemistry (ICC)

ALC cells cultured on chamber slides (Solarbio, China) were transduced with LV-*Odaph* as described. Following PBS rinses, cells were fixed with cold methanol (−20°C, 10 min) and blocked sequentially with: (i) 5% normal goat serum (ZSGB-Bio, China; 30 min) and (ii) avidin/biotin blocking reagents (Vector Laboratories; 15 min each). Primary antibodies were incubated overnight at 4°C: anti-ODAPH (1:200; Proteintech, #91246), anti-LAMC2 (1:100; Invitrogen, #PA5−79578), and anti-ITGB6 (1:200; Proteintech, #28378–1-AP). After PBS washes, specimens were incubated with biotinylated goat anti-rabbit IgG (1:200; Proteintech, #SA00004−2; 1 h, RT), followed by ABC Elite reagent (Vector Laboratories) and DAB development (ZSGB-Bio). Counterstaining with Mayer’s hematoxylin and mounting with neutral gum preceded light microscopic analysis (Olympus BX53F).

### Co-Immunoprecipitation (Co-IP) assay

The pEGFP-C1-ODAPH-Flag and pcDNA3.1-Lamininγ2-His plasmids (constructed by Hedgehogbio, China) were transformed into *E. coli* DH5α competent cells (Takara, Japan) and purified using the EndoFree Mini Plasmid Kit II (TIANGEN BIOTECH, China). HEK-293T cells were transfected with these constructs or empty vector controls using Lipofectamine 3000 (Thermo Fisher Scientific) per manufacturer’s instructions. After 48 h, cells were lysed in ice-cold WB/IP buffer (Beyotime, China) containing protease inhibitors. Lysates were divided for: (1) input controls (20% lysate mixed 4:1 with 5 × loading buffer) and (2) immunoprecipitation (80% lysate incubated with anti-Flag magnetic beads at 4°C overnight). Precipitated proteins were eluted in 1 × loading buffer and analyzed by western blot.

### TGF-β1 treatment and mineralization assay

ALC cells were divided into two experimental groups: (1) acute treatment with 1 ng/mL recombinant TGF-β1 (Novoprotein, China) for 6 h, and (2) chronic exposure to 1 ng/mL TGF-β1 in mineralization medium (containing 10 mM β-glycerophosphate, 50 μg/mL ascorbic acid, and 10 nM dexamethasone) for 7 or 14 days. Untreated cells served as controls. Medium was refreshed every 48 h during prolonged cultures.

### Integrin inhibition assay

ALC cells were treated with either: (1) 1 mM CWHM-12 (MedChemExpress, USA) or (2) vehicle control (0.1% DMSO; Meilunbio, China) for 3 h to assess acute inhibition effects. For chronic inhibition studies, cells were maintained in mineralization medium (containing 10 mM β-glycerophosphate, 50 μg/mL ascorbic acid, and 10 nM dexamethasone) supplemented with 10 μM CWHM-12 for 7 or 14 days, with medium changes every 48 h.

### Statistical analysis

All data were analyzed using GraphPad Prism 9.0 and are presented as mean ± SD from three independent biological replicates. Between-group comparisons were performed using two-tailed Student’s t-tests. A p-value <0.05 was considered statistically significant.

## Results

### *Odaph* overexpression enhances ALP expression

**Fig 1:**
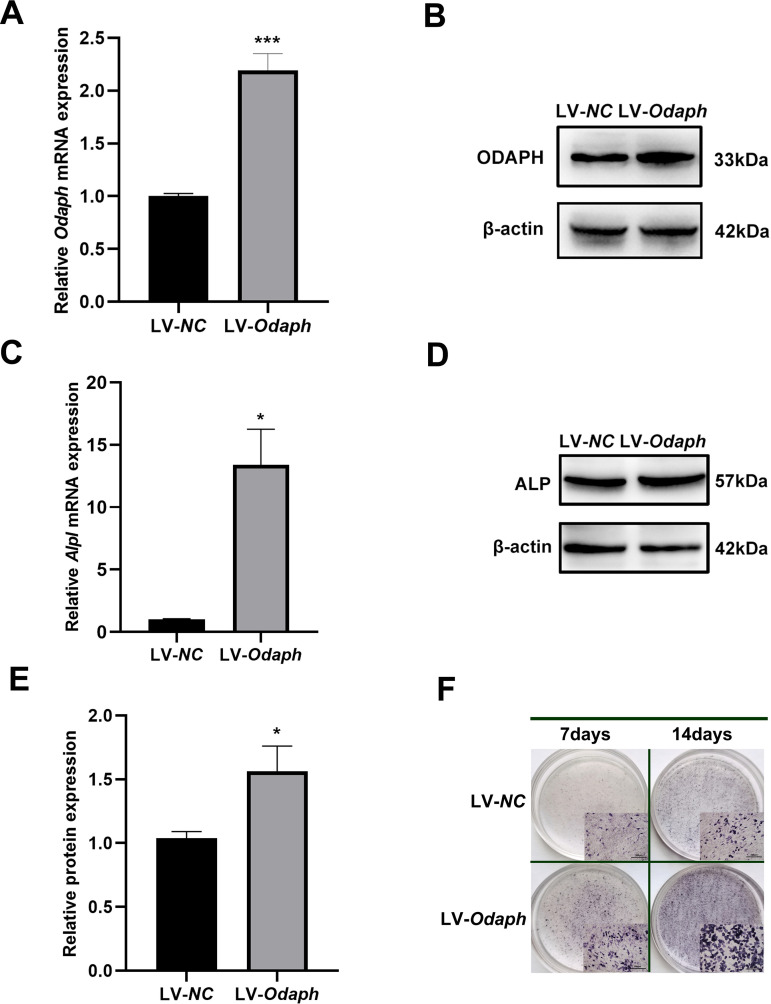
*Odaph* overexpression enhances ALP expression during ameloblast mineralization. (A) qRT-PCR analysis of *Odaph* mRNA levels in: LV18-NC ameloblasts (LV-*NC,* transfected with empty vector lentivirus LV18-NC); ODAPH-overexpressing cells (LV-*Odaph*, transfected with pCMV-ODAPH) after 72-hour transfection. (B) Western blot of ODAPH protein expression in LV-*NC* and LV-*Odaph* groups (β-actin as loading control). (C) qRT-PCR analysis of *Alpl* mRNA levels at day 7 of mineralization induction (DMEM-F12 + 50 μg/mL ascorbic acid, 10 mM/L β-glycerophosphate and 10 nM/L dexamethasone). (D) Representative western blot of ALP protein expression at day 7 of mineralization induction. (E) Densitometric quantification of ALP protein levels normalized to β-actin. (F) ALP staining (BCIP/NBT, purple) in: LV-*NC* and LV-*Odaph* groups at days 7 and 14 of mineralization induction. Insets show 10 × magnified views of stained ameloblasts. Scale bars = 300 μm. *P < 0.05; ***P < 0.001.

To investigate ODAPH’s role in enamel mineralization, we established *Odaph*-overexpressing ALC cells via lentiviral transduction. qRT-PCR and western blot analyses confirmed successful *Odaph* overexpression at 72 h post-transduction (Fig 1A and 1B). Following 7-day mineralization induction, both mRNA and protein levels of ALP were significantly elevated in *Odaph*-overexpressing cells compared to controls (Fig 1C-E). ALP enzymatic activity, assessed by ALP staining, demonstrated time-dependent intensification in ODAPH-overexpressing groups at both 7- and 14-day timepoints (Fig 1F).

### *Odaph* overexpression upregulates LAMC2/ITGB6/TGF-β1 signaling

**Fig 2:**
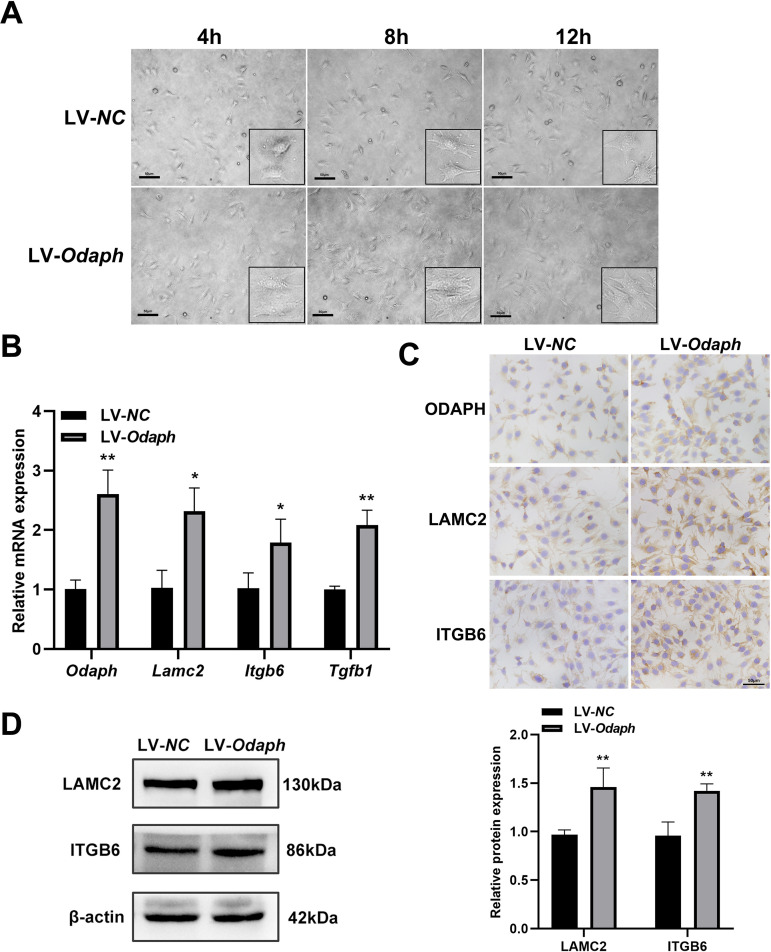
Odaph overexpression upregulates LAMC2/ITGB6/TGF-β1 signaling and promotes ameloblast adhesion. (A) Cell adhesion assay showing morphological changes in: LV18-NC ameloblasts (LV-*NC,* transfected with empty vector lentivirus LV18-NC); ODAPH-overexpressing cells (LV-*Odaph*, transfected with pCMV-ODAPH) at 4, 8, and 12 hours post-seeding. LV-*Odaph* groups exhibited enhanced cell spreading and flattened morphology at 4-8h, with convergence to control morphology by 12h. Insets show 40 × magnified views. (B) qRT-PCR analysis of *Lamc2*, *Itgb6* and *Tgfb1* mRNA levels in LV-*NC* vs. LV-*Odaph* groups at 72h post-transfection. (C) Immunocytochemistry (ICC) of LAMC2 and ITGB6 in ameloblast-lineage cell (ALC) showing cytoplasmic localization. *Odaph* overexpression enhanced expression of both proteins. (D) Western blot analysis of LAMC2 and ITGB6 protein expression in LV-*NC* vs. LV-*Odaph* groups. Relative protein expression was measured by Image J. Scale bars = 50 μm. *P < 0.05; **P < 0.01.

ALC cells transduced with LV-*Odaph* exhibited enhanced adhesion capacity, demonstrating significantly improved cell spreading and flattened morphology at 4–8 h post-seeding compared to controls (Fig 2A). By 12 h, this morphological difference was no longer apparent. At the molecular level, *Odaph* overexpression upregulated: (i) *Lamc2* and *Itgb6* mRNA expression and (ii) downstream *Tgfb1* expression (Fig 2B). Immunocytochemical analysis revealed cytoplasmic localization of LAMC2 and ITGB6, with expression intensities markedly enhanced by *Odaph* overexpression (Fig 2C). Western blot quantitative analysis confirmed the increase of LAMC2 and ITGB6 at the protein level (Fig 2D).

### ODAPH interacts with LAMC2

**Fig 3:**
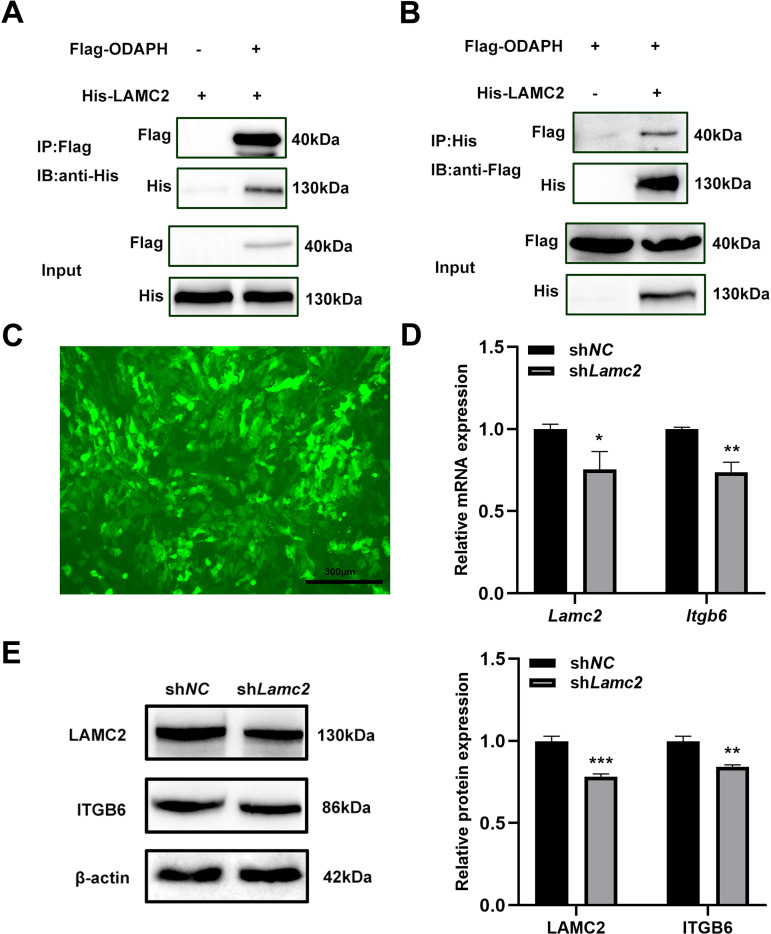
ODAPH physically interacts with LAMC2 and regulates ITGB6 expression. (A, B) Co-immunoprecipitation (Co-IP) analysis of ODAPH-LAMC2 interaction in HEK-293T cells.HEK-293T cells were co-transfected with Flag-ODAPH and His-LAMC2 plasmids. (A) IP performed with anti-Flag magnetic beads, IB with anti-His antibody. (B) Reciprocal IP with anti-His beads, IB with anti-Flag antibody. Input controls shown for both transfections. (C) The transfection efficiency of *Lamc2* knockdown lentivirus was viewed under a fluorescence microscope at 72h post-transduction. (D-E) Effects of *Lamc2* knockdown on ITGB6 expression. (D) qRT-PCR analysis of *Lamc2* and *Itgb6* mRNA levels. (E) Western blot of LAMC2 and ITGB6 protein expression with β-actin loading control. Relative protein expression was measured by Image J. Flag-ODAPH, pEGFP-C1-ODAPH-Flag; His- LAMC2, pcDNA3.1- LAMC2-His. IP, immunoprecipitation; IB, immunoblot. Scale bars = 300 μm. *P < 0.05; **P < 0.01; ***P < 0.001.

Building upon previous reports of ODAPH-LAMC2 co-localization at the basal lamina [9,15]. we performed reciprocal Co-IP assays to validate their molecular interaction. HEK-293T cells were co-transfected with Flag-tagged ODAPH and His-tagged LAMC2 constructs. Immunoprecipitation with anti-Flag beads followed by anti-His immunoblotting revealed specific pulldown of LAMC2 (Fig 3A). Conversely, anti-His immunoprecipitation with anti-Flag detection confirmed ODAPH binding (Fig 3B). These reciprocal experiments demonstrate a direct physical interaction between ODAPH and LAMC2.

### LAMC2 regulates ITGB6 expression

To investigate the functional relationship between LAMC2 and ITGB6, we generated *Lamc2*-deficient ALC cells using GFP-tagged lentiviral shRNA. Fluorescence microscopy confirmed >80% transduction efficiency based on GFP expression. Both qRT-PCR and western blot analyses demonstrated significant downregulation of ITGB6 in *Lamc2*-knockdown cells compared to scramble controls (Fig 3D and 3E). These results establish LAMC2 as a positive regulator of ITGB6 expression.

### TGF-β1 promotes ALP expression during mineralization

**Fig 4:**
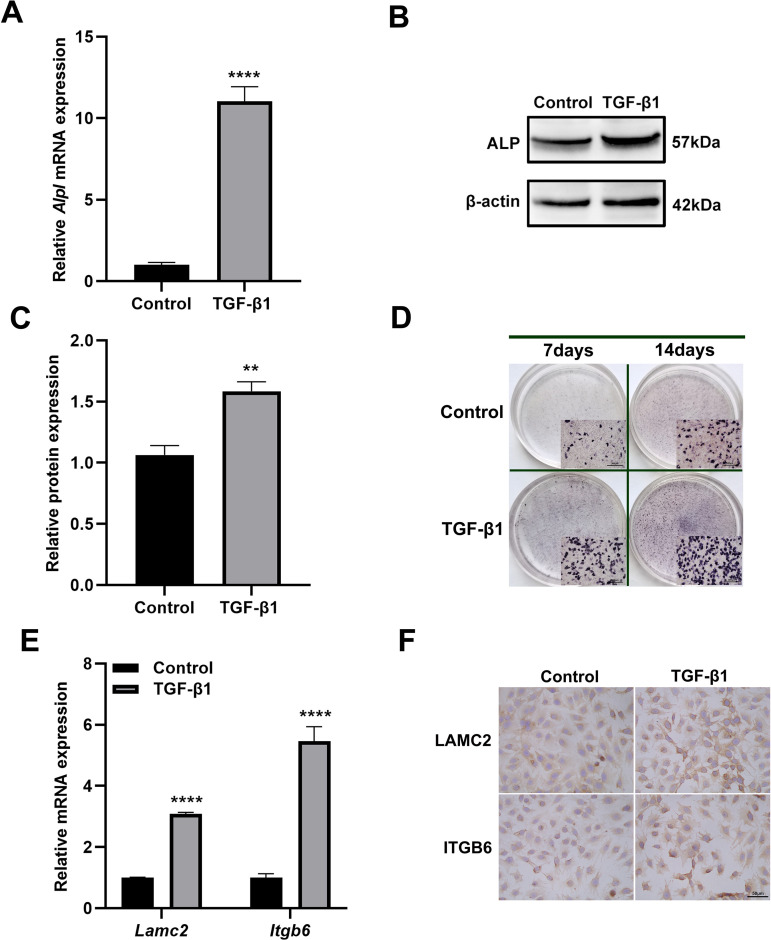
TGF-β1 upregulates ALP, LAMC2 and ITGB6 expression. (A) analysis of *Alpl* mRNA levels in ALC treated with TGF-β1 (1 ng/mL) at day 7 of mineralization induction. (B) Western blot analysis of ALP protein expression under TGF-β1 treatment at day 7 of mineralization induction. β-actin served as loading control. (C) Image J conducted semi-quantitative analysis of the TGF-β1 protein expression. (D) ALP staining (BCIP/NBT, purple) in control vs. TGF-β1 mineralization-inducing groups at days 7 and 14. Insets show 10 × magnified views. (E) qRT-PCR analysis of *Lamc2* and *Itgb6* mRNA levels after 6h TGF-β1 treatment. (F) Immunocytochemistry of LAMC2 and ITGB6, showing enhanced cytoplasmic staining with TGF-β1 treatment. Scale bars, 300 μm in upper panels, 50 μm in lower panels. **P < 0.01; ****P < 0.0001.

ALC cells treated with TGF-β1 (1 ng/mL) demonstrated significantly enhanced ALP expression compared to untreated controls. Both qRT-PCR and western blot analyses revealed that the levels of ALP mRNA and protein increased significantly after 7-day mineralization induction (Fig. 4A-C). Notably, while control groups exhibited expected temporal increases in ALP activity, TGF-β1 treatment consistently produced stronger staining intensities at each matched timepoint (Fig 4D).

### Integrin inhibition attenuates ODAPH-mediated TGF-β1/ALP signaling

**Fig 5:**
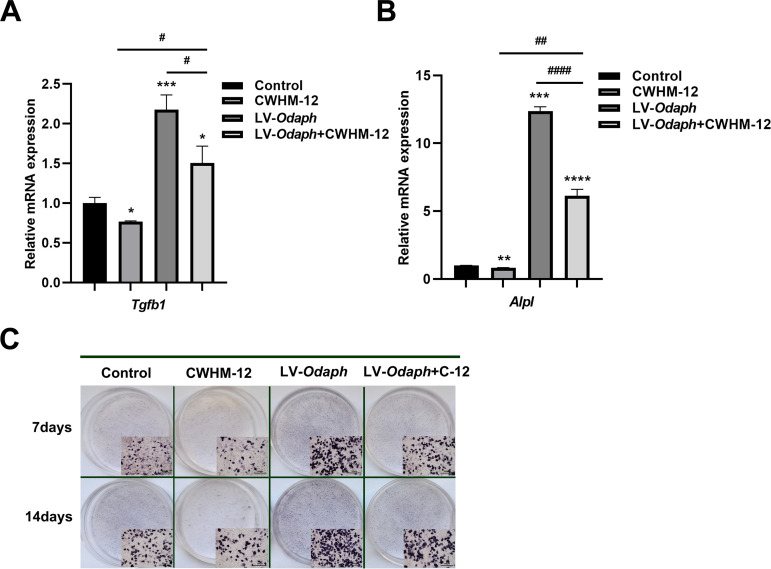
ITGB6 inhibitor CWHM-12 attenuates ODAPH-mediated TGF-β1 signaling and ALP expression in ALC cells. (A) qRT-PCR analysis of *Tgfb1* mRNA levels after 3h CWHM-12 (1 mM/mL) treatment, showing that the addition of CWHM-12 reversed the upregulated expression of *Tgfb1* caused by *Odaph* overexpression. (B) qRT-PCR analysis of *Alpl* mRNA levels at days 7 and 14 of mineralization induction, showing that the upregulated expression of *Alpl* caused by *Odaph* overexpression was reversed after ALC cells were subjected to CWHM-12 (10 µM/mL). (C) ALP staining (BCIP/NBT, purple) at days 7 and 14 of mineralization induction, showing lower ALP staining density in LV-*Odaph*+CWHM-12 groups compared with the *Odaph* overexpression groups. Insets show 10 × magnified views. LV-*Odaph*+C-12, LV-*Odaph*+CWHM-12. Scale bars = 300 µm. *P < 0.05; **P < 0.01; ***P < 0.001; ****P < 0.0001. ^#^*P* < 0.05; ^##^*P* < 0.01; ^####^*P* < 0.0001.

To elucidate ODAPH’s regulatory mechanism, we employed CWHM-12, a specific integrin αvβ6 inhibitor, to block downstream signaling in ALC cells. qRT-PCR analysis revealed three key findings: (1) CWHM-12 significantly reduced basal *Tgfb1* expression; (2) *Odaph* overexpression increased *Tgfb1* expression; and (3) CWHM-12 completely abolished this *Odaph*-induced *Tgfb1* upregulation (Fig 5A). Similarly, during mineralization, CWHM-12 not only decreased basal *Alpl* expression but also reversed the *Alpl* expression induction by *Odaph* overexpression (Fig 5B). CWHM-12-treated groups exhibited markedly reduced ALP staining intensity at both 7- and 14-day timepoints compared to *Odaph*-overexpressing cells (Fig 5C), with the CWHM-12 monotherapy group showing minimal enzymatic activity.

### TGF-β1 upregulates adhesion biomarkers

Complementing its effects on ALP expression, TGF-β1 treatment (1 ng/mL) significantly enhanced the expression of key adhesion molecules. qRT-PCR analysis showed significant increases in *Lamc2* and *Itgb6* mRNA levels (Fig 4E). Immunocytochemical analysis confirmed these findings at the protein level, demonstrating TGF-β1-induced intensification of both LAMC2 and ITGB6 immunoreactivity in the ALC cell cytoplasm (Fig 4F). These results establish TGF-β1 as a positive regulator of ameloblast adhesion machinery.

## Discussion

**Fig 6:**
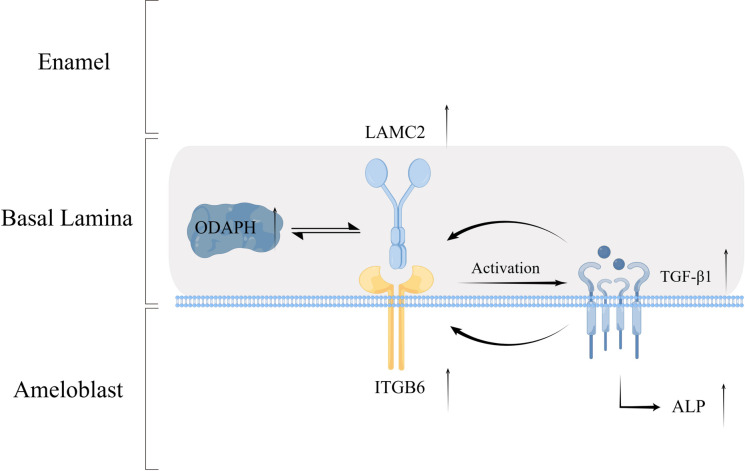
Proposed mechanistic model of ODAPH-mediated ameloblast adhesion and ALP expression. ODAPH associates with LAMC2 and ITGB6 at the ameloblast-enamel interface. This tripartite complex facilitates cell-matrix attachment to developing enamel. The ODAPH-LAMC2-ITGB6 interaction triggers TGF-β1 signaling. TGF-β1 upregulates ALP expression. The molecular mechanism that ODAPH promotes enamel mineralization was further clarified from the perspective of extracellular matrix-mediated attachment of epithelial cells to hard tissues.

Our study delineates a novel ODAPH-LAMC2/ITGB6-TGF-β1 signaling axis critical for ameloblast adhesion and mineralization (Fig 6). LAMC2, as a marker of the atypical basal lamina, was localized in the densa layer of the basal lamina [36]. This distribution of LAMC2 ensures the integrity of the basal lamina and affects the biological behavior of various epithelial cells. Our published study showed that ODAPH and LAMC2 were co-localized on the basal lamina, and expression of LAMC2 was remarkably reduced and disorganized in *Odaph*^*-/-*^ mice, with irregular and diffuse expression between the flattened epithelial cells and the enamel surface [9,15]. Our data demonstrate that *Odaph* overexpression enhanced LAMC2/ITGB6/TGF-β1 pathway activity (Fig 2). This genetic perturbation-rescue logic indirectly supports the functional necessity of the ODAPH-LAMC2 interaction. While our current data strongly suggest a functional interaction, we acknowledge that direct proof would be ideal. Such experiments are technically challenging due to the lack of commercially available tools targeting ODAPH’s binding domain, but we have included this as a key future objective.

It is known that integrin interacted with laminin ligands, mediating the adhesion of epithelial cells to the extracellular matrix [19,37]. ITGB6, as part of the integrin, may be involved in regulating the signaling between the ameloblast and the extracellular matrix, thus affecting various aspects of enamel formation. Our research found that knockdown of *Lamc2* decreased ITGB6 expression in ALC cells (Fig 3). ODAPH might scaffold LAMC2 and ITGB6, as our Co-IP shows, forming a ternary complex that potentiates TGF-β1 signaling. This aligns with our data showing ITGB6 inhibition disrupts ODAPH’s effects (Fig 5). Consistent with our in vitro data, *Odaph* deficient mice exhibit disrupted ameloblast-basement membrane adhesion, further supporting ODAPH’s role via LAMC2/ITGB6. Moreover, we propose ODAPH’s phosphorylated residues (e.g., Ser/Thr) may bind LAMC2’s LG domains, similar to integrin-laminin interactions. Future studies could mutate ODAPH phosphorylation sites to validate this.

Loss of *Itgb6* mice and *Tgfb1* gene conditional knockout mice enamel uncovered abnormal mineralization [38–42], which was consistent with *Odaph* knockout mice. In accordance with the findings that blocking integrins with small molecules can hold back TGF-β signal and prevent liver fibrosis in mice [43], our findings confirmed that CWHM-12 inhibited the activation of TGF-β1 by blocking ITGB6 expression, thus affecting the expression of ALP. It is well known that ALP is an early biomarker of mineralization. Therefore, we propose that ODAPH interacted with LAMC2 and ITGB6, which stimulates the formation of integrin-based multiprotein complexes, triggering TGF-β1 signal to promote ALP expression. As one of the most important functional genes in calcification, it promotes mineralization by increasing the local rate of inorganic phosphate and reducing the concentration of extracellular pyrophosphate [30,44]. ALP is the direct transcriptional target of TGF-β1 in ameloblasts, making it a biologically relevant endpoint for our hypothesis. Therefore, we propose that *Odaph* overexpression accelerated the enamel mineralization by increasing early ALP expression. However, in the process of enamel mineralization, WD repeat-containing protein 72 (WDR72) [45], Stromal Interaction Molecule 1 (STIM1), Solute carrier family 24, member 4 (SLC24A4) plays a key role in calcium transport and matrix protein removal [46,47], and whether ODAPH regulates their expression will be further investigated.

We also discovered that TGF-β1 promoted the expression of LAMC2 and ITGB6 (Fig 4E and 4F). In addition to ITGB6 being an activator of TGF-β1, TGF-β1 activity is necessary to maintain ITGB6 expression in epithelial cells, suggesting that there is a mutual positive feedback loop between the ITGB6 and TGF-β1 [19]. Our study indicated that the upregulated expression of LAMC2 and ITGB6 caused by TGF-β1 further enhanced the adhesion of ameloblast to enamel surface.

Although our previous study [11] confirmed the consistency of ALCs in enamel mineralization with in vivo results, yet in vivo enamel formation takes place in an extremely complex and dynamically changing physiological environment involving multiple cell types, growth factors, hormones, and extracellular matrix interactions. These factors are challenging to fully simulate in vitro. In vivo enamel mineralization is precisely regulated by a variety of molecules and pathways, and these regulatory mechanisms might not be fully activated or expressed in vitro culture, leading to disparities in mineralization events. Despite these differences, in vitro study offers valuable insights for understanding the fundamental mechanisms of enamel mineralization and lays the foundation for future in vivo studies. We explored the enamel mineralization mechanism regulated by ODAPH from the perspective of the role of ODAPH in promoting the attachment of ameloblast to enamel. Although the dish can not completely simulate the complex environment of ameloblast in vivo and mineralized hydroxyapatite (HA) surfaces, it can simulate the adhesion behavior of cells on the surface of hard tissues to a certain extent, providing an important reference for subsequent in vivo experiments and clinical applications.

In conclusion, our study reveals the biological function of ODAPH in amelogenesis from the perspective of the adhesion of epithelial cells to hard tissue mediated by the extracellular matrix. Nevertheless, it is requisite to further explore the function of ODAPH in mice and human and the pathogenesis of genotype to phenotype caused by ODAPH mutations.

## Supporting information

S1 File Raw images. (PDF)
