# Supplementary figures and images for: Odontogenesis-associated phosphoprotein (ODAPH) Promotes Ameloblast adhesion and alkaline phosphatase (ALP) expression via LAMC2/ ITGB6/TGF-β1 signaling pathway
Source: PLoS One. 2025 Jul 18;20(7):e0328263. doi: 10.1371/journal.pone.0328263 (PMC12273980; doi:10.1371/journal.pone.0328263)

## S1\_raw\_images

**Figure 1B:**

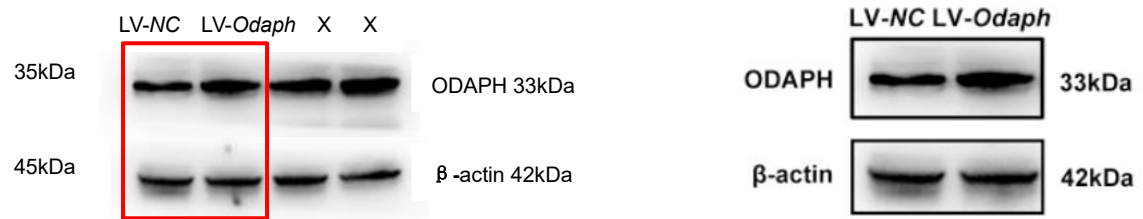

**Figure 1D:**

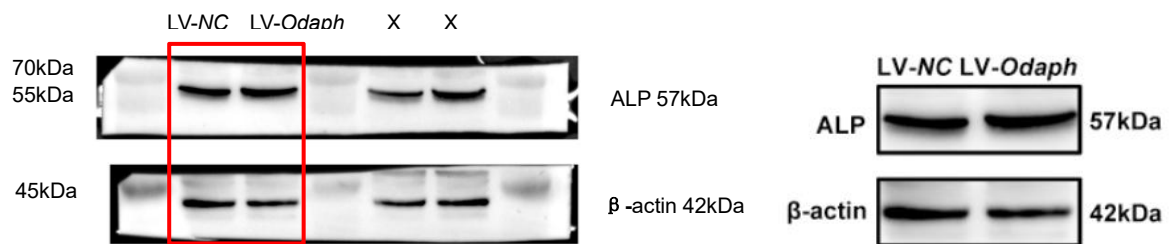

**Figure 2D:**

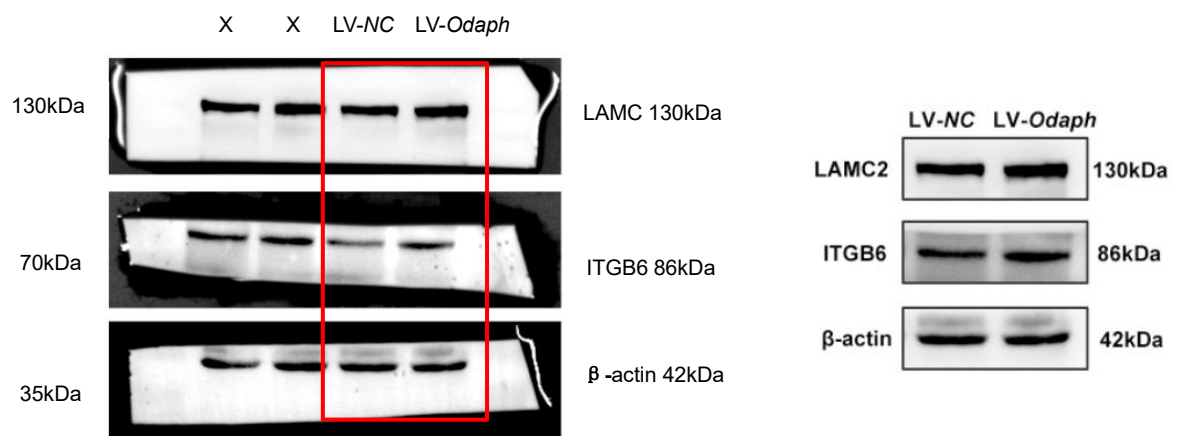

**Figure 3A:**

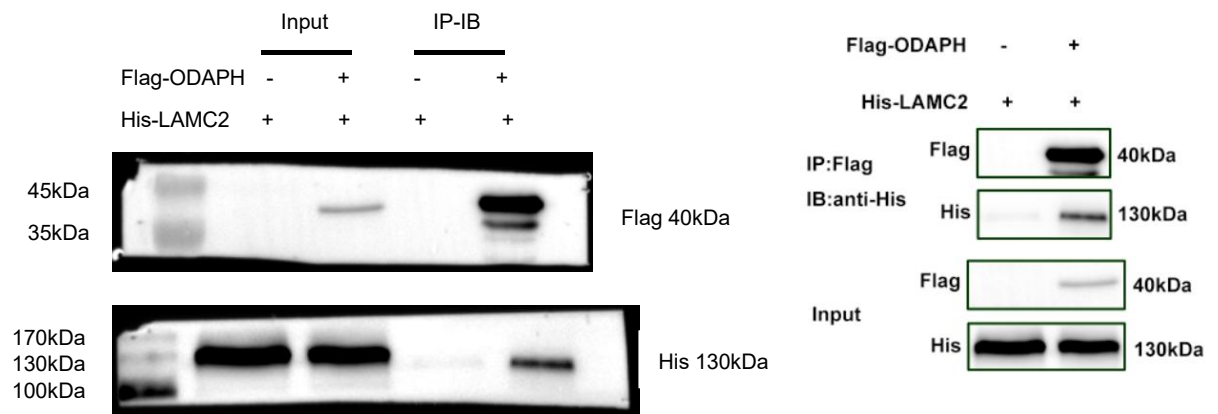

**Figure 3B:**

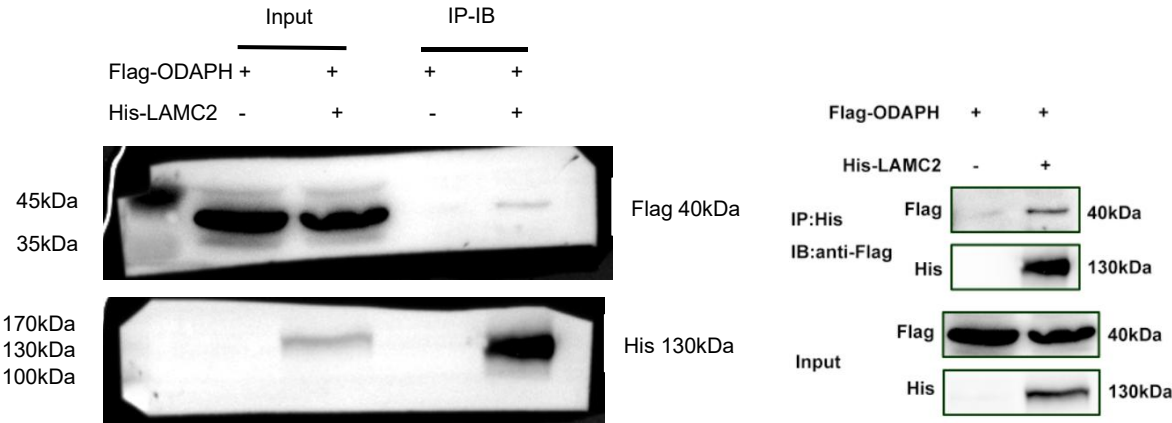

**Figure 3E:**

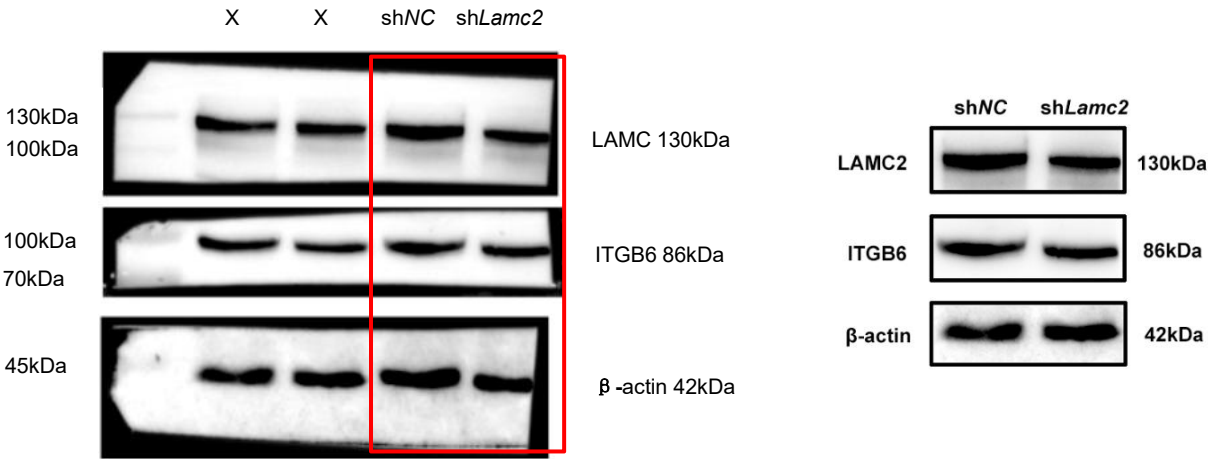

**Figure 4B:**

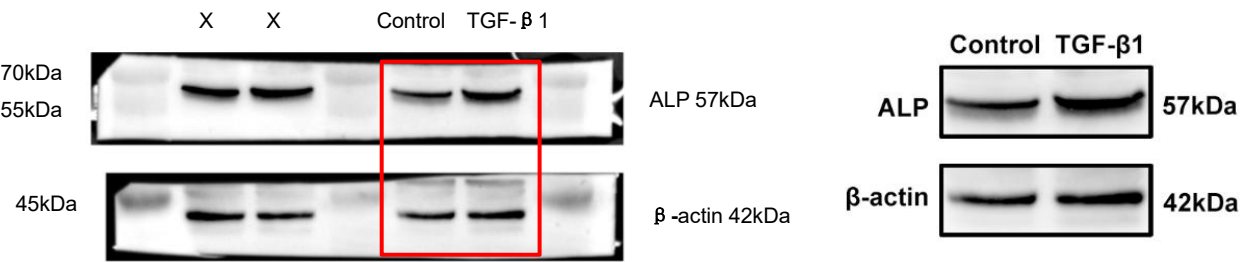

Supplement: S1 File — (PDF) [file pone.0328263.s001.pdf]
